# Use of Dalbavancin in Skin, Bone and Joint Infections: A Real-Life Experience in an Italian Center

**DOI:** 10.3390/antibiotics10091129

**Published:** 2021-09-19

**Authors:** Lucia Brescini, Filippo Della Martera, Gianluca Morroni, Sara Mazzanti, Maria Di Pietrantonio, Paolo Mantini, Bianca Candelaresi, Francesco Pallotta, Silvia Olivieri, Valentina Iencinella, Sefora Castelletti, Emanuele Cocci, Rosaria G. Polo, Salvatore Veccia, Oscar Cirioni, Marcello Tavio, Andrea Giacometti

**Affiliations:** 1 Infectious Diseases Clinic, Ospedali Riuniti Umberto I, Via Conca 71, 60126 Torrette, AN, Italy; filippodellamartera@live.it (F.D.M.); sara.mazzanti@hotmail.it (S.M.); mariadipietrantonio@gmail.com (M.D.P.); paolomantini90@gmail.com (P.M.); b.candelaresi@gmail.com (B.C.); pallottafrancesco1993@gmail.com (F.P.); silviaolivieri1992@gmail.com (S.O.); salvatore.veccia@ospedaliriuniti.marche.it (S.V.); oscar.cirioni@ospedaliriuniti.marche.it (O.C.); Andrea.Giacometti@ospedaliriuniti.marche.it (A.G.); 2 Department of Biomedical Sciences and Public Health, Polytechnic University of Marche Medical School, Via Tronto 10/a, 60020 Torrette, AN, Italy; g.morroni@pm.univpm.it (G.M.); valentinaiencinella2@gmail.com (V.I.); 3 Infectious Diseases, Ospedali Riuniti Umberto I, ViaConca 71, 60126 Torrette, AN, Italy; seforacastelletti@gmail.com (S.C.); marcello.tavio@ospedaliriuniti.marche.it (M.T.); 4 Hospital Pharmacy, Ospedali Riuniti Umberto I, Via Conca 71, 60126 Torrette, AN, Italy; Emanuele.Cocci@ospedaliriuniti.marche.it (E.C.); RosariaGerarda.Polo@ospedaliriuniti.marche.i (R.G.P.)

**Keywords:** dalbavancin, ABSSSI, prosthetic joint infections, osteomyelitis

## Abstract

Dalbavancin is a lipoglycopeptide approved for the treatment of acute bacterial skin and skin structure infections (ABSSSI). The aim of the study was to evaluate the efficacy and safety in all patients who received at least one administration of dalbavancin. Methods: We carried out a retrospective study of the use of dalbavancin in 55 patients at the Azienda Ospedaliera Ospedali Riuniti Umberto I (Ancona, Italy) from February 2017 to May 2020 and compared “on label” and “off label” use of dalbavancin in ABSSSI and non-ABSSSI. Results: A total of 55 patients were included in the study. The median age was 61 years; 51% had ABSSSI; 24% had prosthetic joint infections, and 14% had osteomyelitis. A total of 53% received a single 1500 mg infusion of dalbavancin, and 18% received a second dose 14 days later; 24% of patients received further doses at 14-day intervals. In 91% of cases, patients achieved clinical objectives with dalbavancin: 96% of patients with ABSSSI and 69% of those with prosthetic joint infections. Conclusions: Dalbavancin was shown to have an excellent tolerability profile and to be a highly successful therapeutic approach even in those cases treated “off-label”.

## 1. Introduction

Dalbavancin is a new lipoglycopeptide approved by the European Medicines Agency (EMA) and by the US Food and Drug Administration (FDA) for the treatment of acute bacterial skin and skin structure infections (ABSSSI) in adults [1,2]. It has a spectrum of activity against Gram-positive bacteria, also including drug-resistant isolates, such as methicillin-resistant *Staphylococcus aureus* (MRSA) [3,4]. The particular characteristic of this antibiotic is its 14.4-day half-life and good bone penetration [5,6,7,8] and its excellent tolerability profile [2,7,9]. In ABSSSI, a regimen of two 1000 mg doses on day 1 followed by 500 mg on day 8 and a single 1500 mg administration have both been approved [2,10,11]. Another therapeutic regimen reported in the literature and used in our hospital is a 1500 mg dose on day 1 followed by a further 1500 mg on day 8 [11,12,13]. Several studies of the “off-label” use of dalbavancin have recently been published. In particular, this antibiotic is being used for outpatient parenteral antimicrobial therapy (OPAT) and in infections, such as endocarditis and osteomyelitis, that require long-term antibiotic therapy and long periods of hospitalization [11,12,14,15,16]. The “off-label” use of dalbavancin and the utility of OPAT has also been described in patients classified as vulnerable or high-risk for complications (persons who inject drugs or those who lack social support) [17].

The aim of this retrospective, observational study was to evaluate the efficacy (seen as clinical response at 30 days after administration of the drug) and tolerability of dalbavancin in a hospital in Central Italy. A secondary aim was to compare “on-label” and “off-label” use.

## 2. Results

### 2.1. Patients’ Characteristics, Infection and Microorganisms

The study included a total of 55 patients who had received at least one dose of dalbavancin from February 2017 to May 2020 at the Ospedali Riuniti Umberto I in Ancona, Italy. Characteristics and clinical condition of the patients included in the study are shown in Table 1. Sixty-two percent of patients were male with a median age of 61 years. Most had been admitted to general medical wards (85%) and presented comorbidity, the most frequent being cardiovascular (62%). The median Charlson Comorbidity Index was 3.

Dalbavancin was prescribed for acute bacterial skin and skin structure infections (ABSSSI) in 51% of patients, for prosthetic joint infections in 24%, osteomyelitis in 14%, endocarditis in 2% and septic arthritis in 9%. Most ABSSSI were post-operative wound infections (39%), followed by erysipelas (36%).

Of the 13 patients with prosthetics, 5 had had hip replacements, and 8 had had knee replacements. Time between joint replacement surgery and start of dalbavancin therapy varied from 5 months to 7 years. The number of administrations of dalbavancin also varied (range 1–9).

The most frequently isolated pathogens were MRSA in 16% of cases, *S. epidermidis* in 5% and *E. faecalis* in 4%. A Gram-negative strain was also isolated at the same time in 9% of cases. No pathogen was isolated in 45% of cases. Empirical treatment was given in 54% of ABSSSI patients, in 38% of patients with osteomyelitis and in 46% of patients with prosthetic joint infections. MRSA was found in three ABSSSI patients, in two patients with osteomyelitis, in one patient with prosthetic joint infection, in two patients with septic arthritis and in the only patient with endocarditis. Mixed infections were found in 2 out of 28 cases of ABSSSI, 1 out of 8 cases of osteomyelitis and in 2 out of 13 cases of prosthetic joint infections.

### 2.2. Treatment Characteristics

Treatment characteristics are shown in Table 2. Fifty-three patients had received other antibiotics in the 30 days before administration of dalbavancin. Median time between the previous antibiotic line and start of dalbavancin was 7 days. Piperacillin/tazobactam and teicoplanin were administered in 90% of cases. In patients receiving other treatments, the most common reasons for the switch to dalbavancin were improved patient compliance and a quicker discharge from hospital (49%) and clinical and microbiological failure of the previous antibiotic line (45%).

Dalbavancin was always given at a dose of three 500 mg vials in two hours for a total dose of 1500 mg in each administration. The number of administrations varied from 1 to 9 (median 1; interquartile range [IQR]: 1–9). The interval between administration of each vial also varied. Concomitant antibiotics were used in 20% of cases. The molecules used most often in association with dalbavancin were levofloxacin, ciprofloxacin and cotrimoxazole.

### 2.3. Outcome and Tolerability

**Figure 1:**
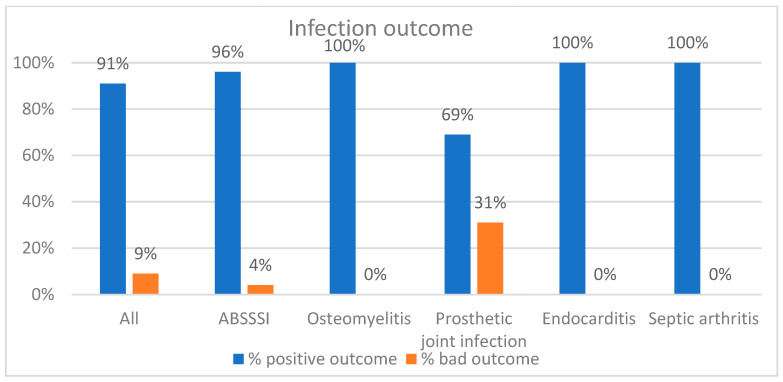
Clinical outcomes after dalbavancin application.

A total of 50 patients (91%) achieved a successful clinical outcome with dalbavancin, seen as improvement or disappearance of signs and symptoms of infection and discharge from hospital (Figure 1). These included 96% of the patients with ABSSSI, 69% of the patients with prosthetic joint infections and 100% of cases of osteomyelitis, endocarditis and septic arthritis.

The characteristics of the five patients who failed therapy are shown in Table 3. Four patients presented with a prosthetic joint infection, and three patients had concomitant Gram-negative bacterial infection.

A slight cutaneous rash was seen in only one patient (Table 2).

### 2.4. Comparison between ABSSSI and Non-ABSSSI Patients

We compared “on-label” and “off-label” use of dalbavancin in two groups of patients: ABSSSI and non-ABSSSI (Table 4). Of the 55 patients analyzed, 28 had ABSSSI, and 27 had other types of infection (non-ABSSSI). There was a statistically significant difference in median age between ABSSSI (56 years; IQR: 52–73) and non-ABSSSI (67 years; IQR: 52–73) patients (*p* = 0.04). Patients with Charlson Comorbidity Index ≥3 were compared for comorbidities and their distribution. These involved 18 out of 28 (64%) cases of ABSSSI and 22 out of 27 (81%) of non-ABSSSI cases, although the difference was not statistically significant (*p* = 0.15). There was a statistically significant difference in median white blood cell count between ABSSSI (8050; IQR: 5610–9197) and non-ABSSSI (6220; IQR: 5645–9147) cases (*p* = 0.02). There was also a statistically significant difference regarding the hospital ward to which the patients were admitted. Among ABSSSI patients, eight (29%) were on the surgical ward, and 20 (71%) were on a general medical ward. All 27 non-ABSSSI patients were admitted to a general medical ward (*p* = 0.007).

A further statistically significant difference was seen in the number of administrations of dalbavancin. ABSSSI patients received a median 1 (IQR: 1–2) administration of the drug compared to a median of 2 (IQR: 1–2) in the non-ABSSSI group (*p* = 0.0002).

Finally, 27 out of 28 (96%) ABSSSI patients achieved a successful clinical outcome and were discharged; only one (4%) patient failed therapy with persistence of signs and symptoms of infection.

Of the 27 non-ABSSSI patients (prosthetic joint infections, osteomyelitis, endocarditis, spondylodiscitis and septic arthritis), 23 (85%) had a positive outcome, while four (15%) failed therapy (three cases of persistent infection, and one patient had the prosthetic joint replaced). However, the difference was not statistically significant (*p* = 0.96).

## 3. Discussion

Dalbavancin is a long-acting antibiotic approved for the treatment of ABSSSI [2]. Its pharmacokinetic characteristics make it an interesting option for Gram-positive infections, such as endocarditis and osteomyelitis, that require long periods of treatment [10,11,12]. Here we describe our clinical experience with dalbavancin from February 2017, both in ABSSSI and in “off-label” use to treat other types of infections. In our case series, frequency of use of dalbavancin was similar in both “on-label” and “off-label” settings. This is in agreement with other European retrospective studies in which this antibiotic was used above all to treat non-ABSSSI [10,14,18]. In contrast, another multicenter observational Italian study that was published recently reported that dalbavancin was mostly used in ABSSSI [17]. In our study, the most frequent non-ABSSSI were prosthetic hip infections (24%), followed by osteomyelitis (14%) and arthritis (9%). Dalbavancin was used in only one patient with endocarditis; this was on completion of another antibiotic treatment before being discharged.

In our study, dalbavancin achieved a high success rate (91% of infections treated) in both ABSSSI (in which it successfully treated the infection in 96% of cases) and in non-ABSSSI infections (85% success rate), without statistically significant differences in efficacy between the two groups. This is in line with other studies reported in the literature in which dalbavancin achieved an overall success rate of 89% [13]. This is higher than that of Bai et al.’s Italian study in which 75% of non-ABSSSI patients were successfully treated [19]. A 100% success rate was achieved in cases of osteomyelitis and septic arthritis, confirming the good bone penetration of this antibiotic [2,10,11,15,20,21]. A recent randomized trial achieved a response rate of 97% in osteomyelitis cases. In this trial, dalbavancin was used as first-line therapy and in the first phase of acute infection [11]. However, Tobutic et al. reported a distinctly lower success rate of 39% in cases of chronic osteomyelitis, thus identifying a different response in cases in which the antibiotic is used in acute infections to those that have already reached the chronic phase [12].

In our case series, we observed a lower success rate in prosthetic hip and knee infections (69%). Prosthetic joint infections are complex infections, due to biofilm formation. Often, these infections require many surgeries, which in some cases do not resolve the associated symptoms [22]. Several in vitro and in vivo studies have assessed the efficacy of dalbavancin against biofilm formation and eradication [22]. However, clinical experiences with dalbavancin for treating prosthetic joint infections were represented by a heterogeneous case series with real-world experience, described in a recent review by Buzòn-Martin et al. [22]. Three major strategies are reported: to attempt eradication and cure with prosthesis retention and debridement; attempt eradication and cure with prosthesis removal; or prosthesis retention (chronic suppressive antimicrobial therapy). This review suggests that the best results are achieved in the case when dalbavancin is combined with adequate surgical source control and prosthesis removal [12,21,22]. Wunsch et al. [13] reported treatment failure in five patients; this was mainly due to inadequate surgical control of the site of infection. On the contrary, the experience of the use of dalbavancin as chronic suppressive therapy is insufficient, and there is little information on the dosage to be used [20]. Infection in our four patients with failure of dalbavancin therapy had been of a longer duration with persistent infection, even with other therapeutic regimens, in line with a therapeutic approach which aims to preserve the prosthesis rather than replace it. In our small sample of patients with prosthetic infections, the number of doses was also very variable (ranging from 1 to 9 doses). In some studies where dalbavancin was used as an immunosuppressive strategy, patients had received up to 20 doses [22]. It would therefore be necessary to have a standardized dosing schedule in this setting of patients. Furthermore, a concomitant Gram-negative bacterial infection was reported in two cases.

In our study, non-ABSSSI patients were significantly older, as they were mostly patients with chronic prosthesis joint infections. In fact, as expected, they also had a lower median blood cell count than the ABSSSI patients. There was also a statistically significant difference regarding the hospital ward to which the patients were admitted: in our case series, all 27 non-ABSSSI patients were admitted to a general medical ward; none of them had been hospitalized in surgical wards. In our experience, and in agreement with clinical trials and real-life reports [10,11,12,13,15,19], dalbavancin demonstrated an excellent tolerability and safety profile, with only one case of slight erythema.

In 53% of cases, dalbavancin was administered in a single 1500 mg dose; this was used in most cases of ABSSSI and septic arthritis. In 18% of cases, a 2-step regimen was used with an initial 1500 mg dose followed by another 1500 mg on day 14; in 24% of cases, this dose was repeated to up to a maximum of nine doses, mostly in cases of prosthetic joint infection.

The most frequent reasons for prescribing dalbavancin in our hospital were failure of previous therapy lines and to improve patient compliance and shorten the hospital stay. Most patients had received another initial antibiotic treatment before being switched to dalbavancin to promote earlier discharge. The Italian study by Bai et al. also showed that 80% of patients had received previous antibiotic therapies [19]. In this study, 70% of ABSSSI patients had received previous antibiotic therapies despite the fact that dalbavancin had been approved as a first-line antibiotic in such infections. Furthermore, 50% received concomitant therapy. The authors considered this a constant cause of concern given the risk of relapse probably related to the residual erythema/edema in ABSSI [19]. In our study, only 20% of the patients received other therapy concomitant to dalbavancin, and most of these were affected by prosthetic joint infections.

## 4. Methods

We conducted this retrospective study considering the period from February 2017 to May 2020 at the Azienda Ospedaliera Ospedali Riuniti Umberto I in Ancona, Italy. The setting was a 980-bedded University Hospital in Central Italy that includes five intensive care units (ICUs), 11 medical and 11 surgical wards. It involved patients aged >18 years who had received at least one dose of dalbavancin and who had been admitted to the hospital over the study period.

Data were collected from the medical case sheets and the laboratory and radiology data available on the hospital’s electronic database. The following variables were considered: patient data (demographics, information concerning chronic and acute comorbidities), information concerning hospital admission (type of infection, microbiological agent responsible, ward to which the patient had been admitted, total length of hospital stay) and treatment-related data.

On completion of treatment, all patients with ABSSSI underwent a 30-days follow up; patients with “off-label” use of dalbavancin (in particular, those with osteomyelitis and prosthetic joint infection) were followed for at least 90 days after the last dose of dalbavancin was administered. In accordance with FDA regulations, ABSSSI are defined as bacterial infections of the skin and soft tissue with a lesion >75 cm^2^ in diameter.

Treatment efficacy was defined by the presence of the following factors: normalization of laboratory tests (C-reactive protein, normalization of the white count), the disappearance of signs and symptoms of infection (erythema, swelling, pain, absence of fever) and the resolution of radiographic signs of infection. Treatment failure was defined as persistence of signs and symptoms of infection at the end of therapy or relapse within 60 days of the last dose of dalbavancin, prosthetic replacement in patients with prosthetic joint infections, interruption of dalbavancin due to toxicity and death. Adverse events were defined as any adverse drug reaction that presents a temporal relationship with dalbavancin [16,17]. Results were analyzed using commercially available statistical software (SPSS 20.0; IBM, Armonk, NY, USA). Qualitative variables were expressed as the frequency of cases, and median and interquartile range of quantitative variables were calculated. Qualitative variables were compared by univariate analysis using the χ2 and Fisher tests. Quantitative variables were analyzed by the Wilcoxon and the Mann–Whitney U tests. *p* < 0.05 was considered statistically significant, and these variables were included in a multivariate analysis by linear logistic regression.

## 5. Conclusions

This study has some limitations related to its single center, retrospective nature, the statistical heterogeneity and the limited number of patients included in the analysis. In addition, in the case of treatment of prosthetic joint infections, the antibiotic treatment was not associated with the surgical intervention. Further studies to compare our findings with groups of patients in whom the two approaches are combined are therefore needed. In conclusion, dalbavancin was shown to have an excellent tolerability profile and to be a highly successful therapeutic approach even in those cases treated “off-label”.

## Figures and Tables

**Table 1:** Patients’ characteristics involved in the study (*n* = 55).

Characteristic	N
**Age, years, median (IQR)**	61 (52–73)
**Gender**	
Male	34 (62%)
Female	21 (38%)
**Department**	
Medical	47 (85%)
ICU	0 (0)
Surgical	8 (15%)
**Underlying diseases**	
Diabetes mellitus	9 (16%)
Cardiovascular disease	34 (62%)
COPD	6 (11%)
Neurological disease	6 (11%)
Solid-organ malignancy	6 (11%)
Gastrointestinal disease	0 (0)
Hematologic malignancy	9 (16%)
Chronic renal failure	5 (9%)
Liver disease	6 (11%)
Chemotherapy	3 (5%)
Immunosuppressive therapy	3 (5%)
Steroid therapy	6 (11%)
Solid-organ transplant	0 (0)
Bone marrow transplant	2 (4%)
**Acute comorbidity**	0 (0)
Charlson comorbidity index (median IQR)	3 (2–5)
**Infection type**	
Prosthetic joint infection	13 (24%)
ABSSSI	28 (51%)
Osteomyelitis	8 (14%)
Endocarditis	1 (2%)
Septic arthritis	5 (9%)
Pathogens	
MSSA	1 (2%)
MRSA	9 (16%)
Enterococcus faecalis	2 (4%)
Enterococcus faecium	0 (0)
S. epidermidis	3 (5%)
MRSE	1 (2%)
Polimicrobial infection ^a^	6 (11%)
Other species ^b^	8 (15%)
Empirical	25 (45%)

N: number; IQR: interquartile range; ICU: Intensive Care Unit; COPD: chronic obstructive pulmonary disease; MSSA: methicillin-sensitive S. aureus; MRSA: methicillin-resistant Staphylococcus aureus; MRSE: methicillin-resistant S. epidermidis. ^a^ 1 patient (2%) had an infection caused by >1 Gram-positive pathogens, and 5 patients (9%) had a mixed infection (Gram-positive + Gram-negative bacteria). ^b^ Other species: Corynebacterium striatum and Streptococcus mitis.

**Table 2:** Previous antibiotic treatments and characteristics of dalbavancin treatment.

Previous Antibiotic Treatment	N
N (%) patients who had received previous antibiotic treatment	53 (96%)
N antibiotics received before dalbavancin therapy, median (IQR)	1 (1–2)
Days of antibiotic treatment before dalbavancin therapy, median (IQR)	7 (1–13)
Total n days of previous antibiotic treatment, median (IQR)	14 (7–30)
**Reasons for dalbavancin use**	
N (%) clinical and/or microbiological failure of previous antibiotic therapy	25 (45%)
N (%) side effects of previous antibiotic therapy	1 (2%)
N (%) multidrug bacterials	2 (4%)
N (%) poor compliance/early discharge	27 (49%)
**Dalbavancin therapy**	
N (%) 1 × 1500 mg	29 (53%)
N (%) 1 × 1500 mg d1 + d8	3 (5%)
N (%) 1 × 1500 mg d1 + d14	10 (18%)
N (%) other regimens (multiple administrations)	13 (24%)
N dalbavancin administrations, median (IQR)	1 (1–2)
N (%) concomitant antibiotic therapy	11 (20%)
N (%) Adverse events	1 (2%)
Outcome	
N (%) successful clinical outcome	50 (91%)
N (%) treatment failure	5 (9%)

N: number; IQR: interquartile range; d: day.

**Table 3:** Characteristics of patients who failed therapy.

Age (Years)	Type of Infection	Microorganism	Number of Administrations	Concomitant Antibiotics	Description
72	PJI	Undetermined	2	No	Knee replacement R
68	PJI	MRSA	1	No	Hip replacement L
59	PJI	Mixed	3	Yes	Hip replacement R
65	PJI	Mixed	9	Yes	Hip replacement R
59	ABSSSI	Mixed	1	Yes	Sternal post-operative wound following myocardial revascularization with Y graft

PJI: prosthetic joint infection; R: right; MRSA: methicillin-resistant *Staphylococcus aureus*; L: left; ABSSSI: acute bacterial skin and skin structure infections.

**Table 4:** Comparison of patients’ characteristics between the acute bacterial skin and skin structure infections (ABSSSI) group and the non-ABSSSI group.

	ABSSSI (*n* = 28)	Other Sites of Infection (*n* = 27)	*p*-Value
**Age, Years, Median (IQR)**	56 (52–73)	67 (52–73)	**0.04**
**Gender (male)**	16 (57%)	18 (67%)	0.47
**Charlson Comorbidity Index ≥ 3**	18 (64%)	22 (81%)	0.15
**WBC, ×10^9^/L, median (IQR)**	8050 (5610–9197)	6220 (5645–9147)	0.02
**CRP, mg/L, median (IQR)**	3 (1.2–5.17)	3 (1.07–5.12)	0.46
**Ward** Surgical Medical ICU	8 (29%) 20 (71%)	0 (0%) 27 (100%)	**0.007**
**Length of hospital stay,** days (median IQR)	15.5 (10–38.5)	24 (10–38)	0.87
**Previous antibiotic therapies**	27 (96%)	26 (96%)	0.97
**N of days of previous antibiotic therapies**	10 (8–30)	15 (7.5–30)	0.18
**N of days of antibiotics before start of dalbavancin therapy (median IQR)**	5.5 (1–12)	10 (1–13)	0.39
**N of dalbavancin administrations (median)**	1 (1–2)	2 (1–2)	**0.0002**
**Concomitant antibiotic therapy**	5 (18%)	6 (22%)	0.78
**Outcome** Successful clinical outcome Treatment failure	27 (96%) 1 (4%)	23 (85%) 4 (15%)	0.96

IQR: interquartile range; WBC: white blood cell count; CRP: C-reactive protein; ICU: Intensive Care Unit; N: number. *p*-values ≤ 0.05 and in bold show statistical significance.

## Data Availability

Data were collected from the medical case sheets and the laboratory and radiology data available on the hospital’s electronic database.
